# Reservoir dynamic evolution and drainage optimization in deep saturated coalbed methane of Shenfu Block

**DOI:** 10.1371/journal.pone.0360289

**Published:** 2026-10-14

**Authors:** Ya juan Wang, Hao Xu, Yi nan Liu, Yan cheng Liu, Heng Wu, Ding Liu, Xiao zhuo Wu, Yu Dong, Xue jing Song, Jin wei Wang

**Affiliations:** 1 School of Energy Resources, China University of Geosciences (Beijing), Beijing, China; 2 China United Coalbed Methane Corp., Ltd., Beijing, China; 3 Researh Institute of Exploration and Development, PetroChina Changqing Oilfield Company, Xi’an, China; 4 Shanxi Coal Geological Investigation and Research Institute CO., Ltd., Taiyuan, China; Henan Polytechnic University, CHINA

## Abstract

China possesses vast deep coalbed methane (CBM) resources, However, the dynamic evolution of reservoir pressure and permeability during the production process remains unclear, which restricts their efficient development. This study conducted to reveal the dynamic evolution of coal reservoir pressure and permeability under different production rates and its influence on the productivity of deep saturated CBM wells, so as to provide a theoretical basis for efficient development. Based on actual production data from deep saturated CBM vertical wells in the Shenfu Block, and considering positive and negative effects on coal reservoirs during drainage, this study utilizes reservoir pressure and permeability prediction models and derives from production dynamics to characterize the variations of reservoir pressure and permeability under different drainage rates, and analyzes their impacts on well productivity. The gas production curves of deep saturated CBM wells in the Shenfu Block can be classified into two types: decline type and stable type. The results show that the initial pressure drop rate is the key factor leading to productivity differentiation. Stable-type wells exhibit a lower initial pressure drop rate, a wider propagation range of reservoir pressure, and a permeability improvement of 5.4%, enabling sustained and efficient CBM production. In contrast, decline-type wells experience an excessively rapid initial pressure drop rate, resulting in slow reservoir pressure decline, limited permeability enhancement of only 0.9%, and a significant drop in productivity at later stages. To achieve high and stable production of deep saturated CBM wells, a strategy of “slow drainage and controlled pressure” should be adopted. It is recommended to control the bottomhole flowing pressure drop rate within 160 kPa/d during the initial production stage; appropriately increase the rate by 50–100 kPa/d during the gas production increase stage to promote gas desorption; and adjust it back to around 50 kPa/d during the later decline stage to maintain production.

## 1. Introduce

In recent years, a series of major breakthroughs have been achieved in the exploration and development of coalbed methane in China, among which deep CBM has become a strategic replacement field for natural gas production growth. Its efficient development is of great significance for optimizing the national energy structure and advancing the “carbon peaking and carbon neutrality” goals [1,2]. In 2023, China’s first deep CBM giant field with reserves exceeding 100 billion cubic meters— the Shenfu Deep Coalbed Methane Giant Field—was discovered at a depth of 2000 m on the eastern margin of the Ordos Basin, with proven geological reserves surpassing 1100 × 10^8^ m³ [3–5]. During the development of deep CBM, the drainage rate is a critical factor affecting productivity. A reasonable drainage regime can maximize the productivity potential of CBM wells [6]. However, an excessively high drainage rate will cause a sharp decline in reservoir pressure near the wellbore. Under the coupled effects of coal matrix shrinkage and increased effective stress, the propagation of the pressure drop funnel is hindered, adversely affecting the productivity of CBM wells [7–10]. Moreover, because deep coal seams are characterized by great burial depth and low original permeability [11], controlling the drainage rate is particularly important.

During the drainage process, the change of drainage performance is manifested macroscopically as fluctuations in gas and water production, whereas microscopically they are reflected in the gas-water migration flow state in the coal reservoir. Investigating the variations in reservoir pressure and permeability during drainage can guide the optimization of the drainage regime and thereby enhance CBM well productivity.In 1993, King [12] first proposed the material balance equation for CBM based on its adsorption–desorption characteristics. Subsequently, Penuela et al. [13] put forward a generalized material balance equation. On this basis, Ahmed et al. [14] further incorporated multiple factors such as initial free gas. Lu Ping et al. [15] introduced the self-regulation effect of coal reservoirs and established a new material balance equation. Regarding permeability models, Gray [16] first calculated the permeability change caused by pressure drop in combination with stress effects in 1987. On the basis of Gray’s work, Harpalani and McPherson [17] constructed an exponential model describing permeability variation with stress, but without considering the change of pore structure. Levine [18] investigated the relationship between fracture width and permeability under various effects from the perspective of fracture width alteration during drainage. Palmer and Mansoori [19] eveloped a permeability model that accounts for changes in cleat porosity. Palmer et al. [20] further established the PMG permeability model, which considers the heterogeneity of cleats and the elastic self-regulation of permeability, based on the original Palmer–Mansoori model. Tang Dazhen et al. [21] built upon the PMG model by incorporating the gas slippage effect, creating a dynamic prediction model of coal reservoir permeability and average reservoir pressure that comprehensively integrates stress sensitivity, pore structure changes, and the slippage effect. This model can simulate the dynamic changes of reservoir pressure and permeability throughout the entire drainage process more comprehensively and accurately; therefore, it was selected as the foundation for this study.

In recent years, scholars have conducted extensive research on the optimization of CBM drainage regimes. Ni Xiaoming et al. [22] fully considered issues related to permeability damage during the initial drainage stage and established a mathematical model of reasonable drainage intensity (dynamic liquid surface decline rate) in that stage. Xie Xueheng et al. [23] studied the relationship between gas production and drainage intensity and found that CBM wells can achieve higher gas production and longer stable production periods with an appropriate drainage intensity. Du Xinfeng [24] carried out triaxial stress permeability experiments on coal samples with different coal structures, combined with actual gas production data of CBM wells, systematically analyzed the dynamic evolution of coal sample permeability during drainage, and proposed a staged pressure-control and stepwise pressure-drop production method. Tian Bofan et al. [25] systematically analyzed the dynamic characteristics of CBM wells in the eastern Yunnan–western Guizhou region, combined with numerical simulations, and suggested that CBM development in this area should follow the principle of “primarily slow drainage” and optimized the initial drainage rate parameters based on simulation results. Zhu Jianying et al. [26] formulated the “522” drainage regime. Based on big data analysis, a quantitative optimization method of drainage regimes for different coal seams with different well types was established. Characterizing reservoir dynamics—particularly the coupled evolution of pressure and permeability under varying extraction schemes—is essential for optimizing field development strategies. For instance, Li et al. [27] developed a multivariate coupled model for enhanced geothermal reservoirs, providing a quantitative framework for evaluating how extraction parameters influence reservoir deformation and energy output. Similarly, Li et al. [28] investigated particle sedimentation behavior in CO_2_ fracturing fluids through a fluid-solid coupling numerical model, revealing the critical control of fluid viscosity and reservoir conditions on fracture conductivity. More relevantly, Li et al. [29] conducted a sensitivity analysis on wellhead stability during hydrate production in the South China Sea, demonstrating that depressurization amplitude and pressure drawdown rate are key factors governing reservoir stability and long-term productivity. These studies collectively underscore the importance of integrating multi-field coupling analysis into the design of drainage schemes for deep coalbed methane reservoirs, where reservoir pressure decline and permeability evolution directly determine production performance.

In summary, although notable progress has been made in CBM drainage regime optimization, the focus has been predominantly on shallow to medium-depth coal seams. The following issues remain in the optimization of drainage regimes for deep saturated CBM wells: deep CBM are characterized by the coexistence of free gas and adsorbed gas, great burial depth, extremely low permeability, and highly complex stress environments. however, Current drainage regimes largely draw upon experience from shallow formations. If the pressure drop rate after fracturing in deep coal seams is too rapid, artificial fractures can easily close due to stress effects; if the drop rate is too slow, free gas is difficult to produce quickly, and the desorption efficiency of adsorbed gas decreases. Nevertheless, there is still a lack of standards for the optimal pressure draopn rate for deep CBM.

Based on this, this study takes the deep saturated CBM in Shenfu block as the object, on the basis of comprehensively considering the coupling effect of the negative effect of effective stress and the positive effect of matrix shrinkage [30], based on the actual production dynamic data, combined with the existing coal reservoir pressure and permeability prediction model, quantitatively characterizes the dynamic changes of reservoir pressure and permeability during the drainage process of deep saturated CBM wells in the block, analyzes the impact of different drainage regimes on productivity, and proposes a drainage regime suitable for deep saturated CBM reservoirs, which provides reference its efficient development.

## 2. Geological settings

The Shenfu Block is located on the eastern margin of the Ordos Basin, straddling two major secondary tectonic units: the Yishan Slope and the Jinxi Flexural Fold Belt. Within the region, the structural deformation is enhanced from west to east. After coal formation, the area underwent collisional orogeny during the Yanshanian period and multiple uplift events during the Himalayan period, resulting in the current structural configuration of an elevated east and a depressed west. This area can be divided from west to east into gentle zone, step-fault zone, and denudation zone, with the target block situated primarily within the gentle zone. The block is a westward-dipping monocline overall, characterized by simple structure and gentle strata. The regional sedimentary environment gradually transitioned from tidal flat–lagoon to fluvial–deltaic facies, during which a variety of peat sedimentary environments developed, giving rise to stable and continuous thick coal seams [31] The No. 8 + 9 coal seam is the main seam in the study area, deposited in barrier island and tidal flat environments. Its burial depth ranges from 1853.5 to 2526.3 m, and coal thickness varies from 3.2 to 22.9 m. Controlled by the westward-dipping structure, the burial depth of the coal seam is greater in the southwest and shallower in the northeast. The No. 8 + 9 coal seam is dominated by primary structure coal, and the maceral lithotypes are predominantly bright coal and semi-bright coal. This study takes the production well data of 11 typical saturated CBM reservoirs in the study area as the main research object. These wells exhibit continuous production, complete geological and production data, drainage duration exceeding one year, and the original reservoir pressure of approximately 20 MPa. Their well location distribution is shown in Fig 1.

**Fig 1:**
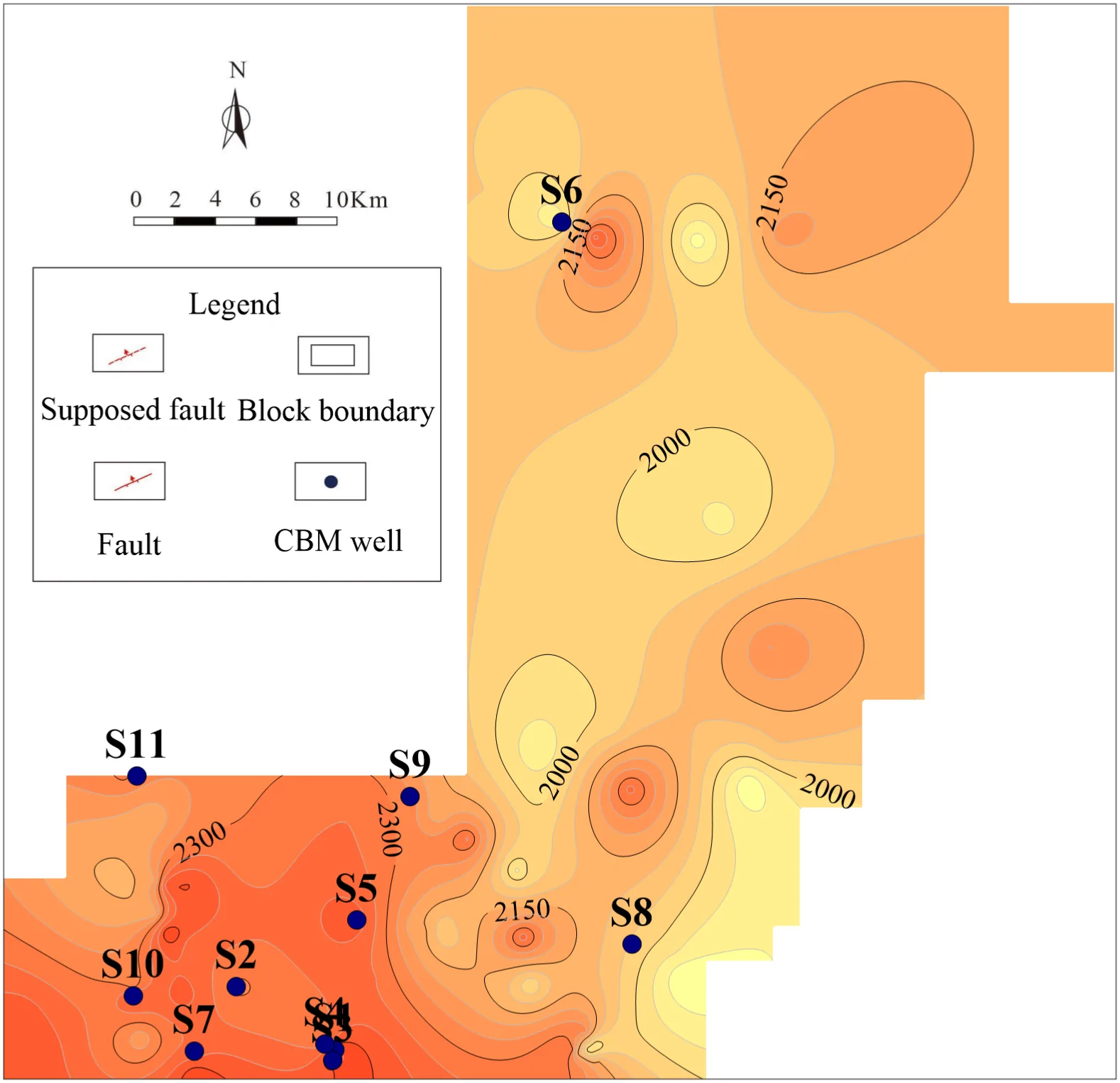
Well location distribution map of Shenfu block in Ordos Basin.

## 3. Methodology

### 3.1. Dynamic reservoir pressure prediction model

A dynamic reservoir pressure prediction model was established based on the dynamic balance mechanism of material and energy [32]. The assumptions of the model are as follows: 1) The coal reservoir is produced under isothermal depletion conditions, with no chemical reaction between the coal and the gas; 2) The CBM reservoir is closed, and boundary-dominated flow will occur in the later stage of production, with through the cracks and follow the radial gas and water seepage of Darcy’s law; 3) The compressibility of the formation water and the rock compressibility are taken into account; 4) Adsorbed gas and free gas coexist within the coal matrix system, and mobile water is present.

In saturated CBM reservoirs, gas–water two-phase flow occurs from the beginning of drainage. The gas–water two-phase material balance equation for the co-flow stage established by Tang Dazhen et al. [21] was selected, and combined with the PMG model, the function for average reservoir pressure is obtained:


$$\begin{array}{c} P=\{\begin{array}{c} -\left[\begin{array}{c} \rho_{B}VV_{L}\frac{{\text{p}}_{\text{0}}}{{\text{p}}_{\text{0}}+{\text{p}}_{L}}+\frac{V{\text{gp}}_{\text{0}}}{MB_{\text{g}}}+\frac{VS_{max}}{3B_{\text{g}}}\left(\frac{1+\nu }{1-\nu }-3\right)\left(\frac{{\text{p}}_{\text{0}}}{{\text{p}}_{\text{0}}+{\text{p}}_{L}}\right)\\ +\frac{V\varphi_{\text{0}}S_{\text{w0}}C_{\text{w}}{\text{p}}_{\text{0}}}{B_{\text{g}}}+\frac{W_{\text{0}}-W_{\text{p}}}{B_{\text{g}}}-G_{\text{p}}-P_{L}\left(\frac{V{\text{gp}}_{\text{0}}}{MB_{\text{g}}}+\frac{V\varphi_{\text{0}}S_{\text{w0}}C_{\text{w}}}{B_{\text{g}}}\right)\\ -\rho_{B}VV_{L}-\frac{VS_{max}}{3B_{\text{g}}}\left(\frac{1+\nu }{1-\nu }-3\right) \end{array}\right]\\ -\left\{ \begin{array}{c} {\left[\begin{array}{c} \rho_{B}VV_{L}\frac{{\text{p}}_{\text{0}}}{{\text{p}}_{\text{0}}+{\text{p}}_{L}}+\frac{V{\text{gp}}_{\text{0}}}{MB_{\text{g}}}+\frac{VS_{max}}{3B_{\text{g}}}\left(\frac{1+\nu }{1-\nu }-3\right)\left(\frac{{\text{p}}_{\text{0}}}{{\text{p}}_{\text{0}}+{\text{p}}_{L}}\right)\\ +\frac{V\varphi_{\text{0}}S_{\text{w0}}C_{\text{w}}{\text{p}}_{\text{0}}}{B_{\text{g}}}+\frac{W_{\text{0}}-W_{\text{p}}}{B_{\text{g}}}-G_{\text{p}}-P_{L}\left(\frac{V\text{g}}{MB_{\text{g}}}+\frac{V\varphi_{\text{0}}S_{\text{w0}}C_{\text{w}}}{B_{\text{g}}}\right)\\ -\rho_{B}VV_{L}-\frac{VS_{max}}{3B_{\text{g}}}\left(\frac{1+\nu }{1-\nu }-3\right) \end{array}\right]}^{\text{2}}\\ -4{\text{p}}_{L}\left(\begin{array}{c} -\frac{V\text{g}}{MB_{\text{g}}}-\\ \frac{V\varphi_{\text{0}}S_{\text{w0}}C_{\text{w}}}{B_{\text{g}}} \end{array}\right){\left[\begin{array}{c} \rho_{B}VV_{L}\frac{{\text{p}}_{\text{0}}}{{\text{p}}_{\text{0}}+{\text{p}}_{L}}+\frac{V{\text{gp}}_{\text{0}}}{MB_{\text{g}}}+\\ \frac{VS_{max}}{3B_{\text{g}}}\left(\frac{1+\nu }{1-\nu }-3\right)\left(\frac{{\text{p}}_{\text{0}}}{{\text{p}}_{\text{0}}+{\text{p}}_{L}}\right)\\ +\frac{V\varphi_{\text{0}}S_{\text{w0}}C_{\text{w}}{\text{p}}_{\text{0}}}{B_{\text{g}}}+\frac{W_{\text{0}}-W_{\text{p}}}{B_{\text{g}}}-G_{\text{p}} \end{array}\right]\}}^{\frac{\text{1}}{\text{2}}} \end{array}\right. \end{array}\}/2/\left(-\frac{V\text{g}}{MB_{\text{g}}}-\frac{V\varphi_{\text{0}}S_{\text{w0}}C_{\text{w}}}{B_{\text{g}}}\right){\text{p}}_{\text{a}}\leq \text{p}\leq {\text{p}}_{\text{0}} \end{array}$$ (1)

Where *P* is the average reservoir pressure, MPa; ρ_B_ is the bulk density of coal, kg/m^3^; V is the gas supply volume controlled by the CBM well, m^3^; V_L_ is the Langmuir volume, m^3^/t; p_0_, the initial reservoir pressure, MPa; p_L_ is the Langmuir pressure, MPa; g is the percentage of horizontal cleats, %; M is the axial modulus, MPa; B_g_ is the gas formation volume factor, m^3^/m^3^; S_max_ is the Langmuir maximum volumetric strain, MPa^−1^; ν is poisson ratio, dimensionless; φ_0_ is the initial porosity, %; S_w0_ is the initial water saturation of the coal reservoir, %; C_w_ is the formation water compressibility, MPa^−1^; *W*_*0*_ is the cumulative encroached volume of edge water, m^3^; *W*_*p*_ is the cumulative water production of the coal reservoir, m^3^; *G*_*p*_ is the cumulative gas production of the coal reservoir, m^3^; p_a_ is the abandonment pressure, MPa.

### 3.2. Reservoir permeability prediction model

Scholars worldwide have conducted extensive research on the dynamic variation of reservoir permeability during the development of CBM wells and have established a variety of models. Currently, the more widely applied models include the P&M model, the CPL model, and others.

Tang Dazhen et al. [21] incorporated the gas slippage effect into the PMG model and developed a new model:


$$\begin{array}{c} \text{k}={\text{k}}_{\text{0}}\left(\frac{1+\frac{\text{b}}{p}}{1+\frac{{\text{b}}_{\text{0}}}{p_{\text{0}}}}\right){\left\{\begin{array}{c} 1+\frac{\text{1}}{\varphi_{\text{0}}}\left[-\frac{g}{M}+\left(\frac{K}{M}+f-1\right)C_{S}\right]\\ \left(p_{\text{0}}-p\right)-\frac{\text{1}}{\varphi_{\text{0}}}\left[\frac{K}{M}-1\right]S_{max}\left(\frac{p_{\text{0}}}{p_{\text{0}}+p_{L}}-\frac{p}{p+p_{L}}\right) \end{array}\right\}}^{\text{3}}p_{a}\leq p\leq p_{\text{0}} \end{array}$$ (2)

Where *k* is the current permeability, mD; k_0_ is the initial reservoir permeability, mD; *b* is the slippage coefficient, MPa; b_0_ is the slippage coefficient at initial reservoir pressure, MPa; *p* is the current reservoir pressure, MPa; p_0_ is the initial reservoir pressure, MPa; φ_0_ is the initial porosity, %; g is the percentage of horizontal cleat porosity, %; M is the axial modulus, MPa; $K=\frac{E}{3\left(1-2\nu \right)}$ is the bulk modulus, MPa; f is a fraction, ranging from 0 to 1; C_s_ is the grain compressibility, MPa^−1^; S_max_ is the Langmuir maximum volumetric strain, MPa^−1^; P_L_ is the Langmuir pressure, MPa_∘_

In this study, based on a prediction model for reservoir pressure and permeability, by inputting the basic parameters shown in Table 1 and importing the actual production data in Table 2, real-time monitoring of the average reservoir pressure and permeability is conducted for 11 deep saturated coalbed methane wells during their development. On this basis, the reservoir response mechanisms under different initial pressure drop rates are analyzed. Due to technical limitations, there are no independent measured reservoir pressure data available for direct model calibration; therefore, the predicted absolute pressure values and permeability enhancement ratios may contain some systematic errors. Nevertheless, the model is built upon the well-established and extensively validated material balance equation and the PMG permeability model, and its predicted relative trends—namely, that slow depressurization facilitates broader pressure drawdown and significantly improves permeability—are physically reasonable.

**Table 1:** Parameters used to simulate the S7 well.

Parameters	acquisition method	value
Coal seam volume/m^3^	Field data	1395000
Initial porosity/%	Sample test report	0.053
Initial water saturation/%	Relative permeability test	0.95
Cleat compressibility/MPa ^− 1^	Laboratory test	0.000325
Formation water compressibility/ MPa ^− 1^	Laboratory test	0.000464
Formation water volume factor/m^3^·m ^− 3^	Laboratory test	1
Grain compressibility/MPa ^− 1^	Laboratory test	0
Gas formation volume facto/m^3^·m ^− 3^	Field data	0.01
Coal density/kg·m ^− 3^	Density test	1430
Langmuir volume/m^3^·t ^− 1^	Isothermal adsorption experiment	18
Langmuir pressure/MPa	Isothermal adsorption experiment	2.5
Abandonment pressure/MPa	Empirical value	0.7
Gas content/m^3^·t ^− 1^	Laboratory test	13.37
Poisson ratio	Triaxial stress test	0.2
Maximum Langmuir volume/ m^3^·t ^− 1^	Isothermal adsorption experiment	0.012
Percentage of horizontal cleat porosity/%	Triaxial stress test	0.1
Young’s modulus/MPa	Triaxial stress test	4000
Axial modulus,/MPa	Triaxial stress test	4800
Slippage coefficient/MPa	Overburden permeability test	0.15

**Table 2:** The actual gas-water production data of the S7 well.

Drainage time/d	accumulated gas production/m^3^	accumulated water production/m^3^	water influx/m^3^
1	214	4.82	0
2	959	9.40	0
:	:	:	:
393	1152234	1154.321	0
394	1153908	1155.281	0

## 4. Results and discussion

For CBM wells in a newly explored block, the universal core control principle of dewatering and gas production is “continuous, slow, long-term, and stable.” Its purpose is to maximize reservoir permeability through controlled pressure drop. Deep saturated CBM reservoirs differ significantly from shallow CBM reservoirs in terms of geological characteristics and development dynamics. Unlike shallow CBM reservoirs, deep saturated CBM reservoirs are characterized by high gas content, high saturation, and abundant free gas. Typically, there is no distinct single-phase water production stage during the drainage process [33]. Gas is often seen after drainage, and the initial stage is two-phase flow. Although this can avoid permeability damage caused by excessive reservoir pressure decline due to prolonged dewatering, the matrix shrinkage effect is weak in the early drainage stage. If an excessively rapid bottomhole flowing initial pressure drop rate is adopted, irreversible damage to the reservoir may still occur. Minimizing the negative effect of effective stress is the key to early-stage drainage.

By using the above models, the dynamic changes in average reservoir pressure and permeability of saturated CBM wells during CBM development can be calculated. Based on the variation characteristics of their gas production curves, the production wells in the study area were classified into two types: decline type and stable type. Considering that the change in bottomhole flowing pressure directly reflects the dynamic variation of coal reservoir pressure near the wellbore and is a direct manifestation of drainage intensity, this study selects the bottomhole flowing pressure decline rate from the start of production until it tends to stabilize (initial pressure drop rate) as the analysis indicator. In this study, based on the production situations of CBM wells in the Shenfu Block, 11 typical wells with continuous and stable production over a period exceeding one year were selected for detailed analysis (Table 3). The results revealed that when the initial pressure drop rate was greater than 160 kPa/d, all wells exhibited a decline type without stable production stage.

**Table 3:** The distribution of well counts with different initial pressure drop rates among various well types.

well type	The number of wells with different pressure drop rate distribution	
	<160 kPa/d	>160 kPa/d
Stage with stable production	6	0
Stage without stable production	0	5

### 4.1. Decline type

For decline-type wells, the bottomhole flowing pressure drops rapidly during the early production stage. Taking Well S2 (Fig 2) as an example, its initial pressure drop rate was as high as 197.3 kPa/d. According to the production data, the well had been producing for a total of 473 days as of the data cutoff, the well was still under production. The gas production rate declined rapidly in the later stage, with an average daily gas production of 3318 m³/d and a relatively low average daily water production of only 4.05 m³/d. Wells of this type exhibit high water production in the early stage, which then drops rapidly in the middle and late stages, and produce almost no water in the later period. Based on the bottomhole flowing pressure curve, the bottomhole flowing pressure declines rapidly in the early stage, and the daily gas production rises quickly, but begins to drop sharply after reaching the peak gas production. Analysis of the average reservoir pressure and permeability curves reveals that as drainage proceeds, the average reservoir pressure declines slowly (from 21.92 MPa to 11.71 MPa), and the permeability improvement is limited, increasing by only 0.9% compared with the initial value, ultimately resulting in relatively low production from the CBM well.

**Fig 2:**
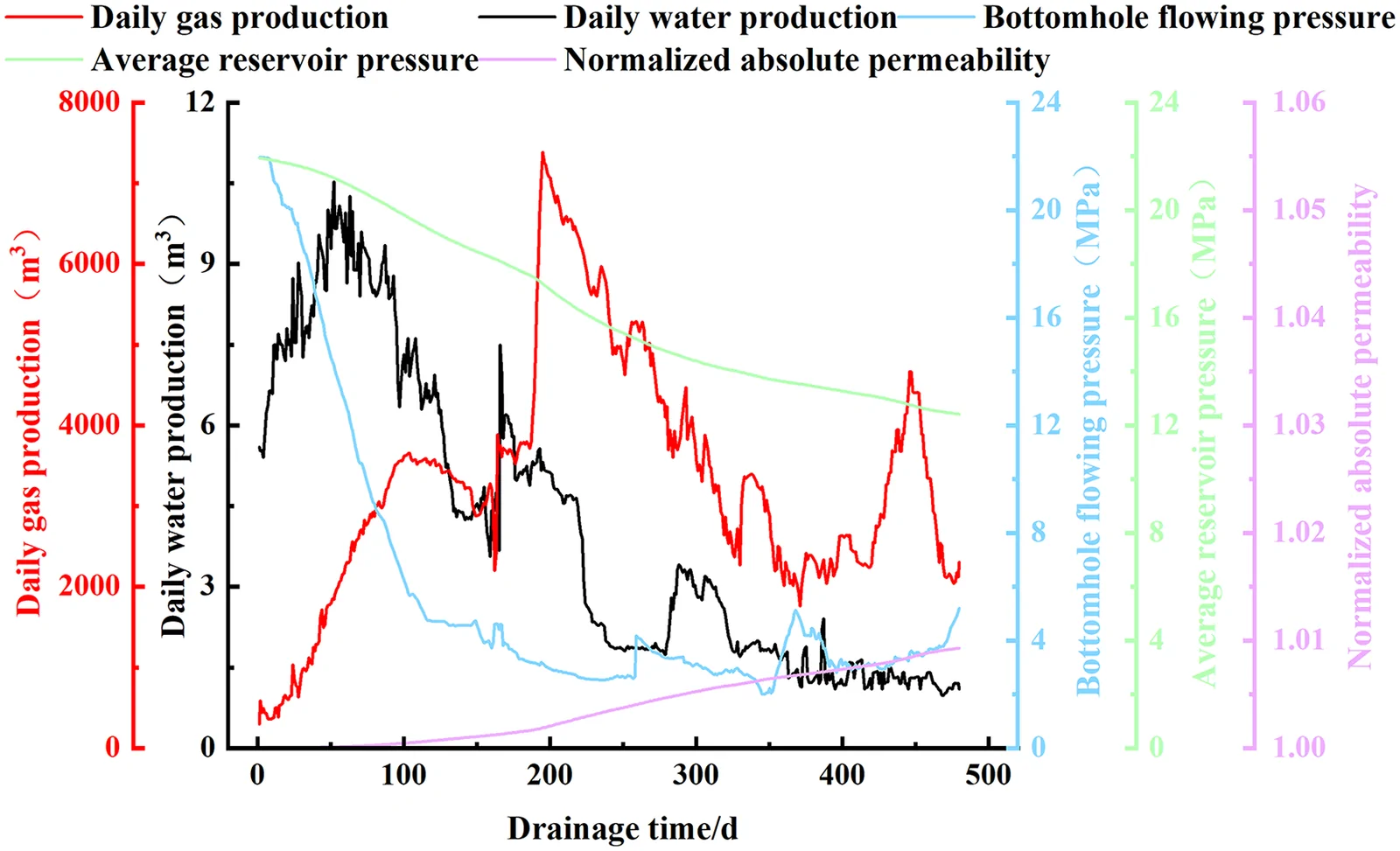
Drainage production curve of a decline-type well.

### 4.2. Stable type

For stable-type wells, the bottomhole flowing pressure exhibits a slow decline trend during the early production stage. Taking Well S11 (Fig 3)as an example, its initial pressure drop rate was 131.14 kPa/d, and the well had been producing for a total of 541 days and as of the data cutoff, the well was still under production. According to the production data, its daily gas production gradually increased in the early period and then stabilized after reaching a certain level, with an average daily gas production of 8073 m³/d and an average daily water production of 2.39 m³/d. The gas production characteristic of this type of well is a continuous increase in the early period, followed by a stable production stage after reaching the peak. The water production characteristic is relatively high water production in the early stage, a gradual decline in the mid-term, and almost no water production in the later stage. As can be seen from the bottomhole flowing pressure curve, the initial pressure drop rate was relatively slow, and gas production peaked when the bottomhole flowing pressure dropped to 10 MPa. Analysis of the average reservoir pressure and permeability curves reveals that the average reservoir pressure decreased from 22.06 MPa to 3.7 MPa, and the permeability increased by 5.4%. Due to the low initial pressure drop rate, the reservoir pressure declined slowly, ensuring a stable expansion of the pressure drop funnel. Meanwhile, the negative effect of effective stress was relatively weak. Moreover, as CBM desorption continued, the coal matrix shrinkage effect gradually intensified, resulting in better permeability performance of the reservoir, which enabled gas production to show a steadily rising trend and maintain stable production after reaching the peak.

**Fig 3:**
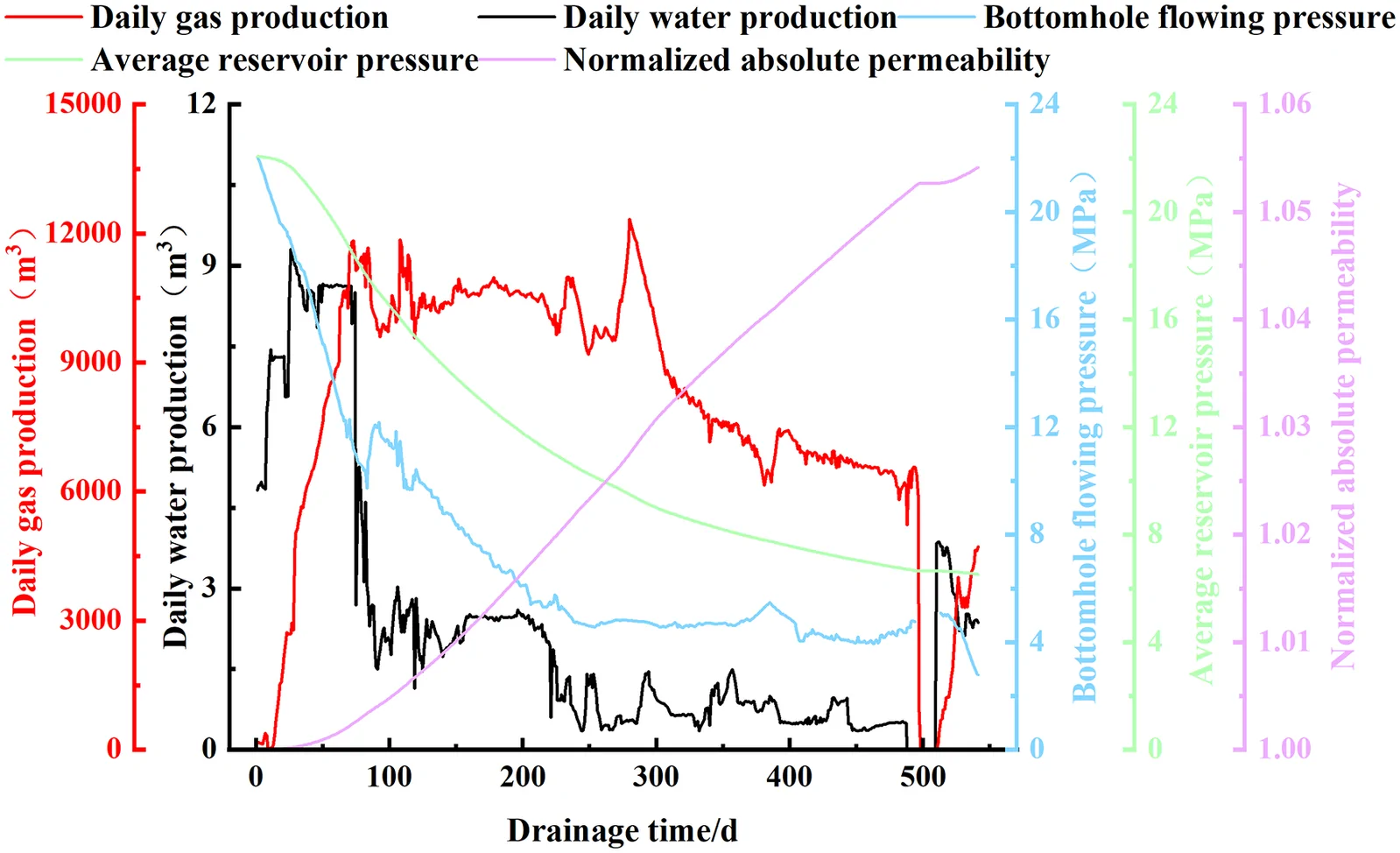
Drainage production curve of a stable- type well.

The drainage rate is the most critical factor affecting productivity within the drainage regime of CBM wells, and its impact is mainly manifested in the early stage of drainage [27].

### 4.3. Comparison of drainage regime

When analyzing the bottomhole flowing pressure curves of stable-type and decline-type wells, geographically adjacent Wells S7 and S3 were selected for comparison to reveal the causes of their productivity differences. Since these two wells are relatively similar in geological conditions and fracturing operation parameters (Table 4), the influence of geological and engineering factors on the productivity difference can be essentially ruled out. Comparing the production curves of the two wells shows that Well S7 exhibited a clear upward trend in daily gas production during the early stage, but it began to decline sharply after a period, whereas Well S3 displayed characteristics of high and stable production.

**Table 4:** Geology and fracturing parameters of Wells S7 and S3.

Well Number	S7 Well.	S3 Well.
Roof	Mudstone	Mudstone
Coal Seam Thickness/m	15.5	16.3
Buried Depth/m	2518	2493
Fracturing Fluid Volume/m^3^	2263.6	2390.1
Fracturing Sand Volume/m^3`^	301.0	321.1
Fracturing Displacement/m^3`^	20.0	20.0

Analysis of the drainage data from the two wells (Fig 4) reveals that their early-stage drainage indicators differed significantly. An analysis of the initial bottomhole flowing pressure variation trends (Fig 4c) shows that Well S7 had an initial pressure drop rate of 245.733 kPa/d, reached a peak gas production of 6301 m³/d after approximately three months of production, and then showed a declining trend. In contrast, Well S3 had a relatively moderate initial pressure drop rate of 117.433 kPa/d. Under the combined effects of effective stress and matrix shrinkage, its daily gas production increased rapidly and gradually stabilized above 10,000 m³/d. In comparison, the initial pressure drop rate of Well S7 was excessively rapid. Although the upward trend in early-stage gas production was more pronounced, the overly rapid initial pressure drop rate adversely affected the reservoir, resulting in insufficient productivity in the later stage and causing a continuous decline in daily gas production.

**Fig 4:**
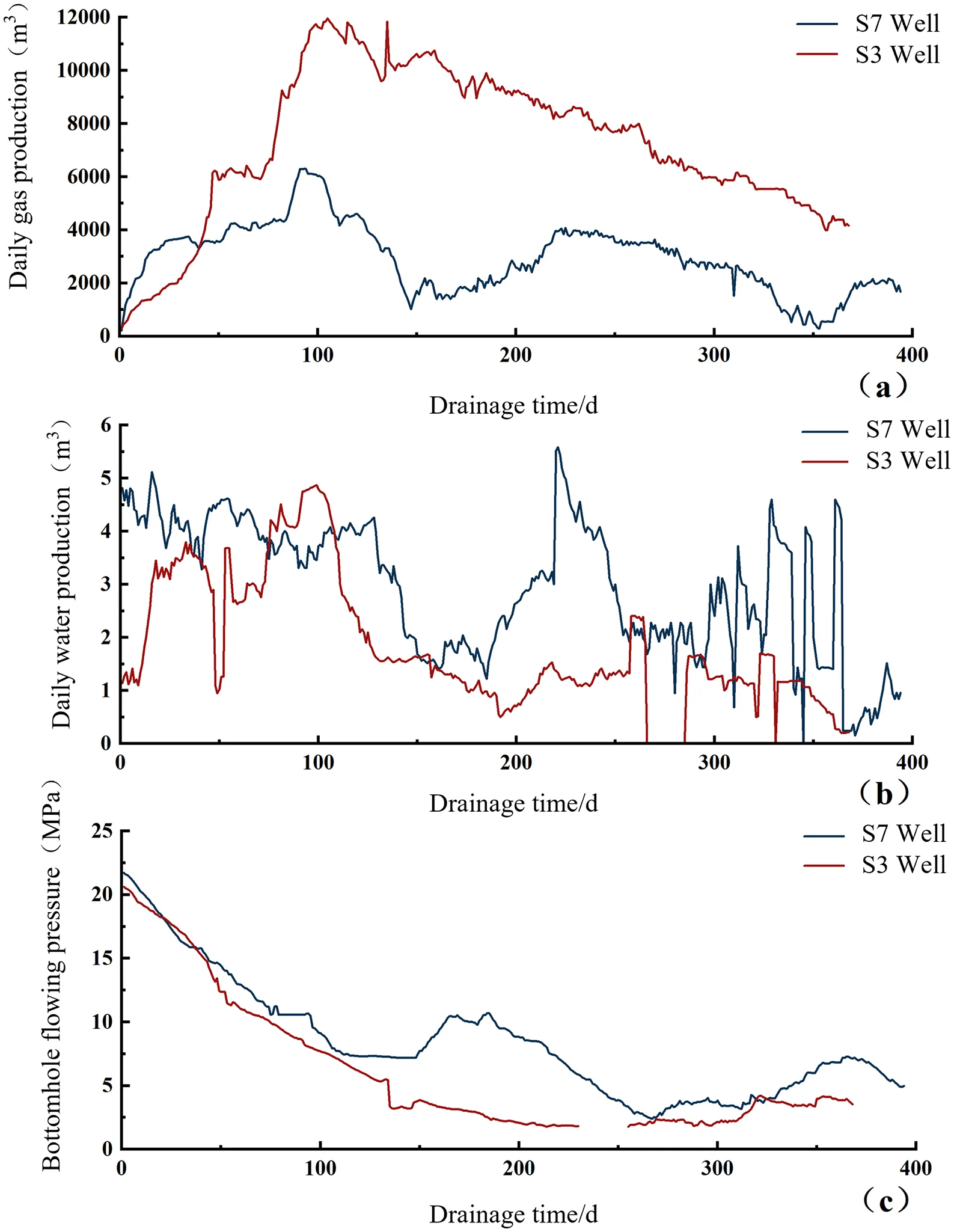
Variation curves of drainage indicators for Well S7 and Well S3 in the Shenfu Block.

### 4.4. Analysis of the causes of gas production decline

In deep saturated CBM wells, free gas dominates during the initial drainage stage. As CBM and coal seam water are continuously produced, the effective stress acting on the coal matrix gradually increases. If the initial pressure drop is substantial, fluids near the wellbore will flow rapidly toward the wellbore under a large pressure differential, causing a sharp pressure decline in the vicinity of the wellbore, while the pressure in regions far from the wellbore declines slowly. This leads to premature fracture closure, resulting in poor permeability improvement in the coal reservoir and restricting the propagation range of the pressure drop funnel [34]. Well S7 was drainaged at an excessively rapid rate during the initial drainage stage. Although its early-stage gas production increased significantly, the excessively rapid pressure drop rate reduced the pore and fracture volume near the wellbore, resulting in poor permeability improvement (the permeability increased by only 0.8% compared to the initial value), and the average coal reservoir pressure decreased from 21.7 MPa to only 15.42 MPa (Fig 5), thereby adversely affecting the high and stable production of the CBM well in the later stage. In contrast, Well S3 adopted a relatively slow initial pressure drop rate. Its average coal reservoir pressure decreased from 20.6 MPa to 11.3 MPa, demonstrating a notable pressure drop effect. The significant difference in reservoir pressure variation between the two wells is mainly attributable to the excessively rapid initial pressure drop rate of Well S7, which prevented the pressure dop funnel from fully expanding, making reservoir depressurization difficult.

**Fig 5:**
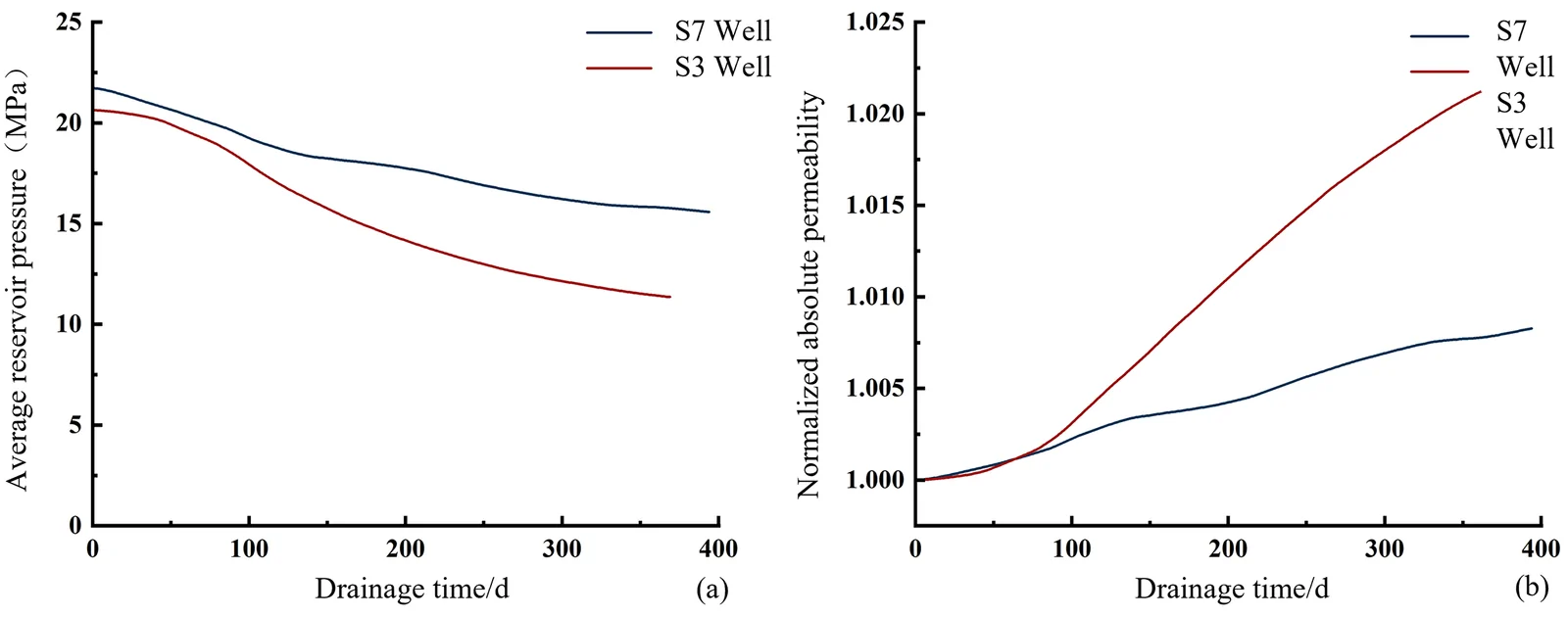
Comparison of average reservoir pressure and permeability between Well S7 and Well S3 in Shenfu Block.

### 4.5. Optimization of the drainage regime

Deep coal reservoirs have low initial permeability, and even a small permeability reduction under effective stress can lead to a significant decline in flow capacity. To intuitively compare the effects of different drainage n regimes, Fig 6 shows the reservoir pressure and permeability changes after 200 days of continuous drainage The simulation results indicate that, under the combined effects of effective stress and matrix shrinkage, an excessively high initial pressure drop rate intensifies the negative effect of effective stress, making reservoir depressurization more difficult, resulting in limited permeability improvement, delayed replenishment by gas desorption, insufficient gas supply, and difficulty in maintaining a stable production trend. In contrast, a moderate initial pressure drop rate facilitates the steady expansion of the pressure drop area, creating a larger effective depressurization zone, thereby ensuring sufficient gas supply and sustaining a stable production trend.

**Fig 6:**
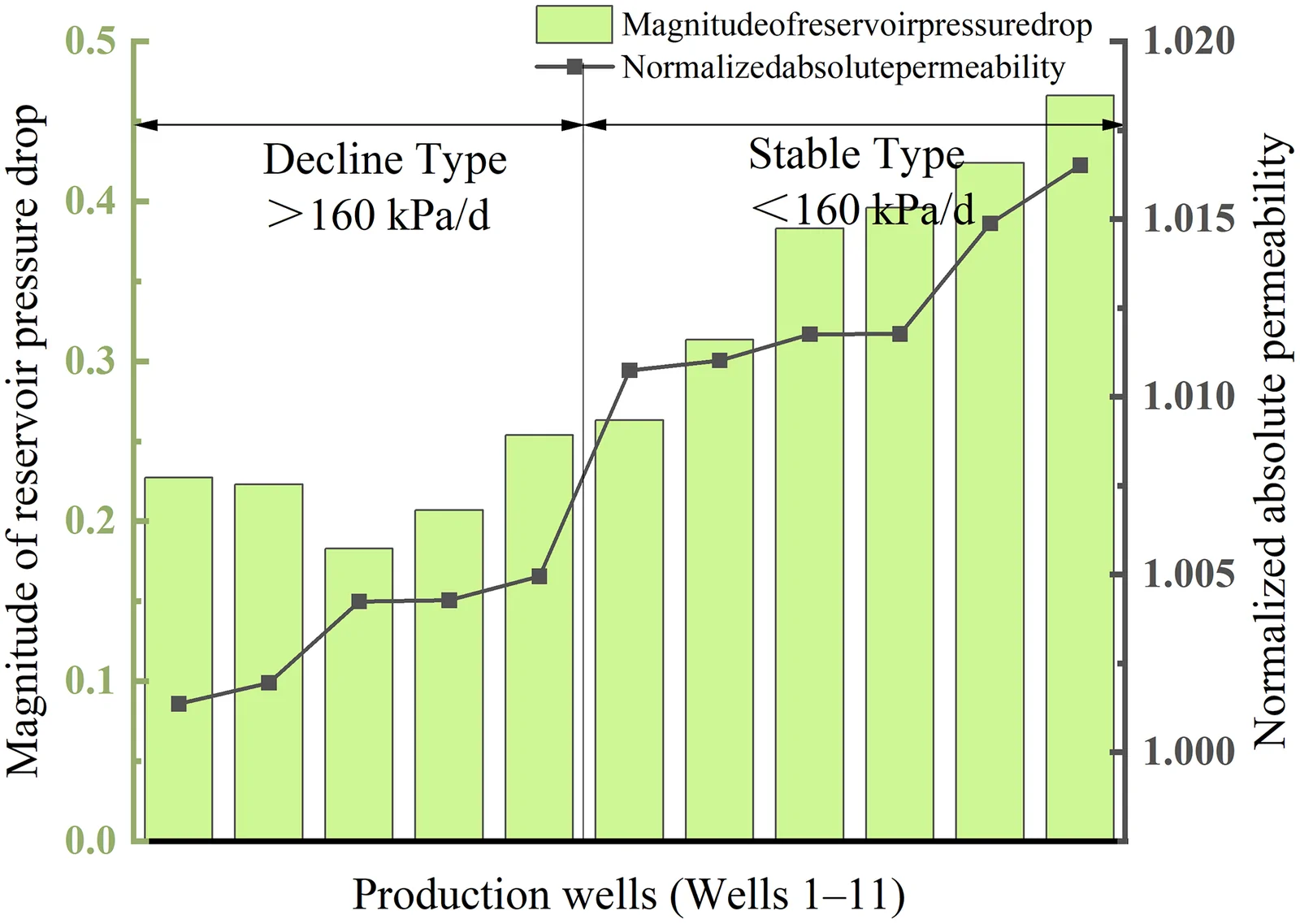
Variations in reservoir permeability and pressure after 200 days of production.

Based on the above analysis, a staged drainage regime is recommended for the deep saturated CBM reservoirs in the Shenfu Block. According to the method for dividing CBM drainage stages, the drainage process of saturated CBM wells can be divided into three stages: the gas production increase stage, the stable gas production stage, and the gas production decline stage. In the Shenfu Block, the initial pressure drop rate should be strictly controlled within 160 kPa/d. After the pressure drop funnel has expanded steadily, the drop rate can be gradually increased. In the later part of the gas production increase stage, the pressure drop rate can be appropriately increased (by 50–100 kPa/d) to accelerate gas desorption and enhance CBM well productivity. In the later part of the decline stage, the production pressure differential should be gradually reduced, with the pressure drop rate controlled at approximately 50 kPa/d, to maintain the production status of the gas well as much as possible. Implementing this staged drainage regime can effectively extend the stable production period, improve the overall development efficiency of CBM wells, and provide a reliable guarantee for continuous and stable production.

The staged drainage regime proposed in this study was applied to a CBM well in the Shenfu Block, and its production curve is shown in Fig 7. After applying this production regime, the well’s peak gas production reached 14,651 m³/d. Although it declined somewhat after reaching the peak, it subsequently entered a high and stable production period lasting over 200 days, with daily gas production stabilized above 9,000 m³/d, demonstrating a pronounced stable production effect. Compared with previous production wells in the same block, this well showed distinct advantages in both stable production duration and productivity stability, thereby validating the effectiveness of the proposed production regime.

**Fig 7:**
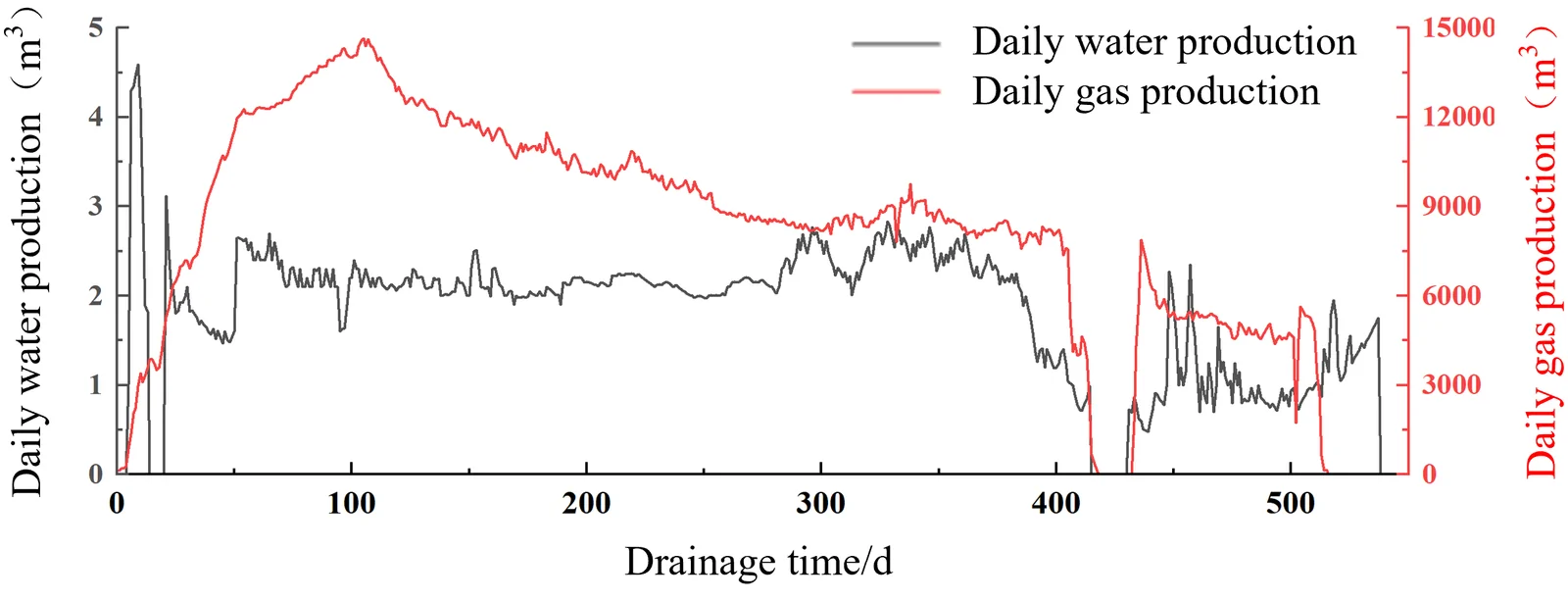
Production curve after the application of the new drainage system.

The core concept of the “slow pressure drop” drainage regime proposed in this study is to balance the positive and negative effects on the reservoir by controlling the pressure drop rate. This concept has universal guiding significance for deep CBM reservoirs. However, the specific optimized thresholds (such as the initial drop rate) need to vary from block to block. When extending this method to blocks with higher in-situ stress and lower permeability, a more conservative initial pressure drop rate is recommended to mitigate the stronger negative stress sensitivity effect. Conversely, for blocks with lower reservoir pressure, the acceleration stage strategy can be appropriately optimized to release production capacity rapidly. Therefore, the key to future application lies in accurately grasping the core geological parameters of the target block (such as in-situ stress gradient, reservoir pressure, reservoir permeability) and, on this basis, conducting targeted optimization using the coupled dynamic simulation method adopted in this study.

## 5. Conclusions

(1) The productivity types (decline type and stable type) of deep saturated coalbed methane wells are essentially controlled by the initial pressure drawdown rate. Rapid pressure drawdown (>160 kPa/d) causes a sharp increase in effective stress near the wellbore, making it difficult for the pressure drop funnel to expand outward and preventing the positive effect of matrix shrinkage from being fully realized. This manifests as almost no permeability improvement, significant differences in the degree of pressure depletion, and rapid productivity decline. In contrast, slow pressure drawdown (<160 kPa/d) facilitates the formation of a large-scale effective depressurization zone, allowing the matrix shrinkage effect to gradually compensate for the negative effect of effective stress, resulting in marked permeability improvement and thereby achieving high and stable production.
(2) For deep saturated coalbed methane wells in the Shenfu Block, a staged production system characterized by “slow pressure drawdown in the early stage, moderate acceleration in the middle stage, and deceleration in the late stage” is recommended. After casing pressure is observed, the initial drawdown rate should be strictly controlled within 160 kPa/d. In the late stage of the production ramp-up period, the drawdown rate can be increased by 50–100 kPa/d to promote gas desorption. During the production decline stage, the drawdown rate should be adjusted to approximately 50 kPa/d to extend the period of stable production. Field application results show that a well adopting this system achieved a peak daily gas production of 14,651 m³ and maintained stable production for over 200 days.
(3) This study is based on the production data of 11 typical wells. The average reservoir pressure and permeability during the production process were all obtained through model calculations. Restricted by technical conditions, these have not yet been independently verified against measured pressure and permeability buildup data, and thus should be interpreted with caution. Additionally, the influences of inter-well interference and complex fracture networks were not considered. In the future, it is advisable to conduct pressure and permeability tests during the production process to calibrate the model, and to couple geomechanical simulations to characterize the stress-seepage field evolution in heterogeneous reservoirs. Meanwhile, a multi-well interference model should be introduced to explore optimized production strategies for cooperative depressurization at the well-pattern scale.

## Supporting information

S1 File Data. (XLSX)
